# Protective Effects of Ferulic Acid on High Glucose-Induced Protein Glycation, Lipid Peroxidation, and Membrane Ion Pump Activity in Human Erythrocytes

**DOI:** 10.1371/journal.pone.0129495

**Published:** 2015-06-08

**Authors:** Weerachat Sompong, Henrique Cheng, Sirichai Adisakwattana

**Affiliations:** 1 Program in Clinical Biochemistry and Molecular Medicine, Department of Clinical Chemistry, Faculty of Allied Health Sciences, Chulalongkorn University, Bangkok, Thailand; 2 Research Group of Herbal Medicine for Prevention and Therapeutic of Metabolic Diseases, Chulalongkorn University, Bangkok, Thailand; 3 Department of Comparative Biomedical Sciences, School of Veterinary Medicine, Louisiana State University, Baton Rouge, Louisiana, United States of America; 4 Department of Nutrition and Dietetics, Faculty of Allied Health Sciences, Chulalongkorn University, Bangkok, Thailand; University of Colorado Denver School of Medicine, UNITED STATES

## Abstract

Ferulic acid (FA) is the ubiquitous phytochemical phenolic derivative of cinnamic acid. Experimental studies in diabetic models demonstrate that FA possesses multiple mechanisms of action associated with anti-hyperglycemic activity. The mechanism by which FA prevents diabetes-associated vascular damages remains unknown. The aim of study was to investigate the protective effects of FA on protein glycation, lipid peroxidation, membrane ion pump activity, and phosphatidylserine exposure in high glucose-exposed human erythrocytes. Our results demonstrated that FA (10-100 μM) significantly reduced the levels of glycated hemoglobin (HbA1c) whereas 0.1-100 μM concentrations inhibited lipid peroxidation in erythrocytes exposed to 45 mM glucose. This was associated with increased glucose consumption. High glucose treatment also caused a significant reduction in Na^+^/K^+^-ATPase activity in the erythrocyte plasma membrane which could be reversed by FA. Furthermore, we found that FA (0.1-100 μM) prevented high glucose-induced phosphatidylserine exposure. These findings provide insights into a novel mechanism of FA for the prevention of vascular dysfunction associated with diabetes.

## Introduction

Chronic hyperglycemia is a major factor in the onset and progress of diabetic complications. Several mechanisms linking hyperglycemia to diabetic complications include the formation of advanced glycation end-products (AGEs), polyol activation, and increased reactive oxygen species (ROS) [1]. Excessive production of ROS leads to oxidative damage and structural/functional alternations to DNA, proteins, and membrane lipids. Human erythrocytes are important for oxygen transport and elimination of carbon dioxide. Likewise, they are highly susceptible to protein and lipid oxidation that can alter the membrane structure and function (e.g. fluidity, permeability, and enzyme activity) [2]. As an example, dysfunction of membrane ion pumps such as Na^+^/K^+^-ATPase pump [3] and lipid peroxidation [4] are directly linked to diabetic vascular complications. Other studies suggest that phytochemical compounds can prevent high glucose-induced erythrocyte membrane damage due to their antioxidant activity [5,6].

Ferulic acid (4-hydroxy-3-methoxycinnamic acid), a cinnamic acid derivative, belongs to a large family of biologically active substances in vegetables [7], fruits [8], and medicinal herbs [9]. Previous studies show that ferulic acid acts as a free radical scavenger such as hydroxyl and peroxyl radicals [10] and an inhibitor of lipid peroxidation [11,12]. Improvement of hyperglycemia in diabetic rats was shown during ferulic acid treatment [13]. In addition, the compound decreases oxidative stress and inflammation in diabetic nephropathy [14]. A number of studies suggest that the anti-hyperglycemic effect of ferulic acid occurs by multiple mechanisms [15,16]. One such mechanism involves inhibition of α-glucosidase and stimulation of insulin secretion [15,16]. Most strikingly, ferulic acid acts as a potent inhibitor of glucose-, fructose-, and ribose-induced protein glycation and oxidative damage in bovine serum albumin (BSA) [17]. Despite the available information, there are no studies examining the effect of ferulic acid on protein glycation in human erythrocytes. The objective of this study was to test whether ferulic acid can reduce protein glycation, lipid peroxidation, and phosphatidylserine exposure and increase Na^+^/K^+^-ATPase activity in erythrocytes under high glucose condition. The findings may provide insights into the mechanism by which ferulic acid prevents vascular and other diabetic related damages.

## Materials and Methods

### Chemicals and reagents

Ferulic acid, butylated hydroxytoluene (BHT), 2-thiobarbituric acid (TBA), malondialdehyde tetrabutylammonium salt, adenosine 5'-triphosphate disodium salt hydrate (ATP-Na_2_), ammonium molybdate, and ascorbic acid were purchased from Sigma-Aldrich Co. (St. Louis, USA). Dimethyl sulfoxide (DMSO) and Trichloroacetic acid (TCA) were obtained from Merck (Darmstadt, Germany). Glucoese oxidase reagent and HbA_1C_ liquidirect reagent were purchased from HUMAN (Wiesbaden, Germany). Bio-Rad protein assay was obtained from Bio-Rad (Hercules, USA). FITC annexin V/dead cell apoptosis kit was purchased from Molecular Probes (Eugene, USA). All other chemicals and solvents were of analytical grade.

### Preparation of human erythrocytes

Whole blood samples were collected from 6 healthy volunteers ages 18–25 non-obese, non-smoker, non-alcohol consumer, and free of any medicines, drugs, or nutritional supplements. Ethylenediaminetetraacetic acid (EDTA) was used as anti-coagulant and blood samples were centrifuged at 1,000 g at 4°C for 10 min. Then, the plasma and buffy coat layers were discarded. The erythrocyte layers were washed three times with cold 150 mM NaCl and centrifuged at 1,000 g at 4°C for 10 min. The supernatants were discarded after each centrifugation. The erythrocyte layers were re-suspended in phosphate-buffered saline (PBS, pH 7.4, containing 1 mM NaH_2_PO_4_, 16 mM Na_2_HPO_4_, and 140 mM NaCl). The Ethics Review Committee for Research Involving Human Research Subjects, Health Science Group, Chulalongkorn University approved the protocol (Protocol number 055.1/55) and consent form. All subjects read and signed a written informed consent before their enrollment into the study.

### *In vitro* treatment of erythrocytes with glucose

Erythrocytes were treated with glucose according to a previous method with minor modifications [18]. The reaction mixtures contained 10% hematocrit erythrocytes and 5 or 45 mM glucose in PBS (pH 7.4) and were incubated at 37°C for 24 h in a shaking incubator. Ferulic acid at 0.1, 1, 10, and 100 μM in 0.1% DMSO were added into the reaction mixtures containing glucose. The final concentration of 0.1% DMSO had no effect in the experiments.

After incubation, the percentages of hemolysis in the reaction mixtures were measured according to a previous method with minor modifications [19]. Briefly, the reaction mixtures were centrifuged at 1,000 g at 4°C for 10 min. The supernatants were measured at 540 nm. The percentage hemolysis was calculated by comparison to 100% hemolysis control (prepared by the incubation of 10% hematocrit erythrocytes with deionized water instead of PBS) using the equation below. The percentages of hemolysis were < 2% in each reaction mixture.

$$\%\;Hemolysis\;=\;\frac{{Abs}_{540\;nm}\;in\;the\;reaction\;mixtures}{{Abs}_{540nm}\;in\;100\%\;hemolysis\;control}\;x\;100$$

Before biochemical analysis, the reaction mixtures were washed three times with cold 150 mM NaCl and centrifuged at 1,000 g at 4°C for 10 min to remove the remaining glucose and ferulic acid. The supernatants were discarded after each centrifugation. Then, the erythrocyte layers were re-suspended in PBS (pH 7.4). During this process, there is some erythrocyte loss in the samples. To normalize the amount of erythrocytes, the levels of hemoglobin (Hb) was determined with Drabkin’s reagent according to a previous method with minor modifications [20]. The erythrocyte samples were adjusted to the same amount of Hb before biochemical analysis.

### Measurement of protein glycation (glycated hemoglobin or HbA_1c_)

The levels of glycated hemoglobin or HbA_1c_, an Amadori product of protein glycation in hemoglobin was used as a marker and determined with HbA_1c_ liquidirect reagent according to manufacturer’s protocol. The erythrocyte samples were lysed with hemolysis reagent and incubated with latex reagent at 37°C for 5 min. The absorbance was measured at 610 nm. The levels of HbA_1c_ were calculated from a standard curve using HbA_1c_ and expressed as %HbA_1c_.

### Measurement of glucose utilization

The levels of glucose utilization were measured according to a previous method with minor modifications [21]. The concentrations of glucose were measured before and after the 24 h incubation period by glucose oxidase reagent according to manufacturer’s protocol. The absorbance was measured at 505 nm. The levels of glucose utilization were calculated by subtracting glucose levels at 24 h from glucose levels at 0 h using the equation below. The result was expressed as mmol/L.

Glucoseutilization(mmol/L)=Glucoselevelsat0h-Glucoselevelsat24h

### Measurement of lipid peroxidation

The levels of malondialdehyde (MDA) were used as a marker for lipid peroxidation using thiobarbituric acid reactive substances (TBARS) according to a previous method with minor modifications [18]. The erythrocyte samples were mixed with PBS (pH 7.4) and BHT (0.88% w/v in ethanol). Then, TCA (30% w/v) was added into the reaction mixtures and kept on ice for 2 h and centrifuged at 2,000 g at 4°C for 15 min. The supernatants were incubated with TBA (1% w/v in 0.05 mM NaOH) and heated in a boiling water for 15 min. After cooling, the levels of TBARS were measured at 532 nm and calculated from a standard curve using malondialdehyde tetrabutylammonium salt. The results were expressed as nmol/mg Hb.

### Measurement of Na^+^/K^+^-ATPase activity

The erythrocyte membranes were prepared according to a previous method with minor modification [22]. Erythrocyte samples were lysed with Tris-HCl (15 mM, pH 7.4) and centrifuged at 12,000 g at 4°C for 30 min. The supernatants were discarded after the centrifugation. The erythrocyte membranes were washed with Tris-HCl (15 mM, pH 7.4) until the color of the membrane pellet was pale. Thereafter, deionized water was added to re-suspend the membranes. The concentration of protein was measured by Bio-Rad protein assay according to manufacturer’s protocol.

The Na^+^/K^+^-ATPase activity was done according to a previous method with minor modifications [23]. The erythrocyte membranes were incubated with reaction buffer A containing 4 mM MgCl_2_, 3 mM ATP-Na_2_, and 50 mM Tris-HCl, pH 7.4, and buffer B containing 120 mM NaCl, 20 mM KCl, 4 mM MgCl_2_, 3 mM ATP-Na_2_, and 50 mM Tris-HCl, pH 7.4 at 37°C for 1 h. After the incubation, the levels of phosphate (Pi) released from ATP-Na_2_ were measured according to a previous method with minor modifications [24]. The reaction mixture was incubated with ammonium molybdate (2.5% w/v) at room temperature for 10 min. Then, ascorbic acid (2% w/v) was added and kept at room temperature for 20 min for color development. The absorbance was measured at 725 nm. The levels of Pi release were calculated from a standard curve using KH_2_PO_4_. Na^+^/K^+^-ATPase activity was calculated using the equation below. The results were expressed as nmole Pi/mg protein/hour.

$${Na}^{+}/K^{+}-ATPase\;activity\;=\;P_{i}in\;the\;reaction\;buffer\;B-P_{i}in\;the\;reaction\;buffer\;A$$

### Measurement of phosphatidylserine exposure

The percentages of phosphatidylserine exposure on the erythrocyte membranes were measured by FITC annexin V/dead cell apoptosis kit according to manufacturer’s protocol. The erythrocyte samples were suspended in annexin-binding buffer and incubated with FITC annexin V at room temperature for 15 min. After incubation, annexin-binding buffer was added and analyzed by flow cytometer at an excitation wavelength of 494 nm and emission wavelength of 518 nm. Annexin V-positive cells were identified as apoptotic cells. The results were expressed as %phosphatidylserine exposure.

### Statistical analysis

The results were expressed as mean±standard error of mean (SEM) (*n* = 6). The statistical significance was evaluated using one-way ANOVA. Tukey’s HSD test was used to determine significant differences between means. *P*<0.05 was considered to be statistically significant.

## Results

### Effects of ferulic acid on protein glycation (glycated hemoglobin or HbA_1c_)

**Fig 1:**
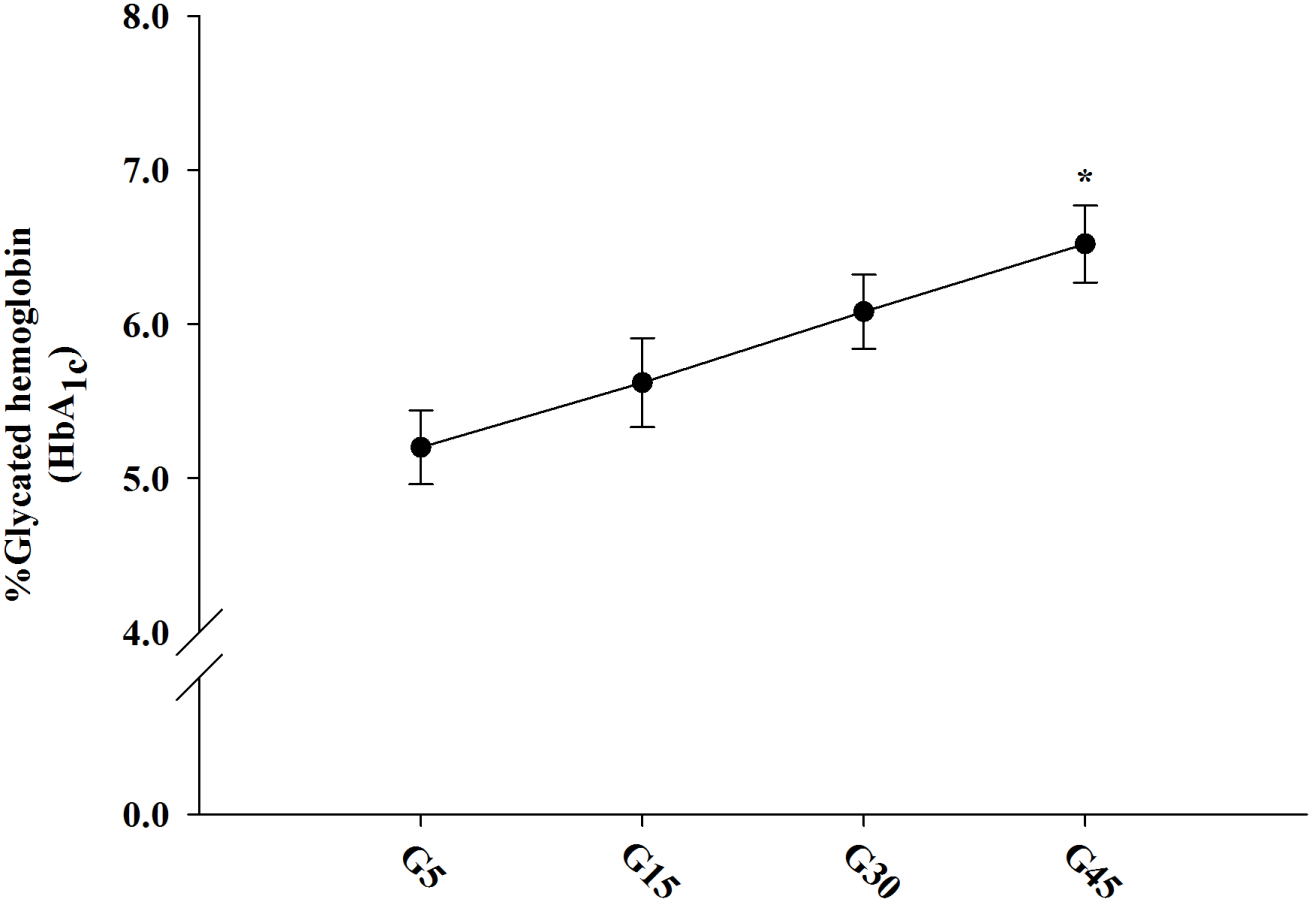
The concentration-dependent effects of 5–45 mM glucose on protein glycation (glycated hemoglobin or HbA_1c_) in erythrocytes at 37°C for 24 h. The results were expressed as mean±SEM (*n* = 6). *p<0.05 when compared to 5 mM glucose (G5) treatment.

The concentration-dependent effects of glucose on Amadori product formation in erythrocytes are shown in Fig 1. After 24 h of incubation, glucose (15–45 mM) increased HbA_1c_ levels ranging from 5.62–6.52%. Erythrocytes treated with high glucose (45 mM) significantly increased the levels of HbA_1c_ compared to 5 mM glucose. Therefore, we selected the 45 mM concentration for further experiments.

**Fig 2:**
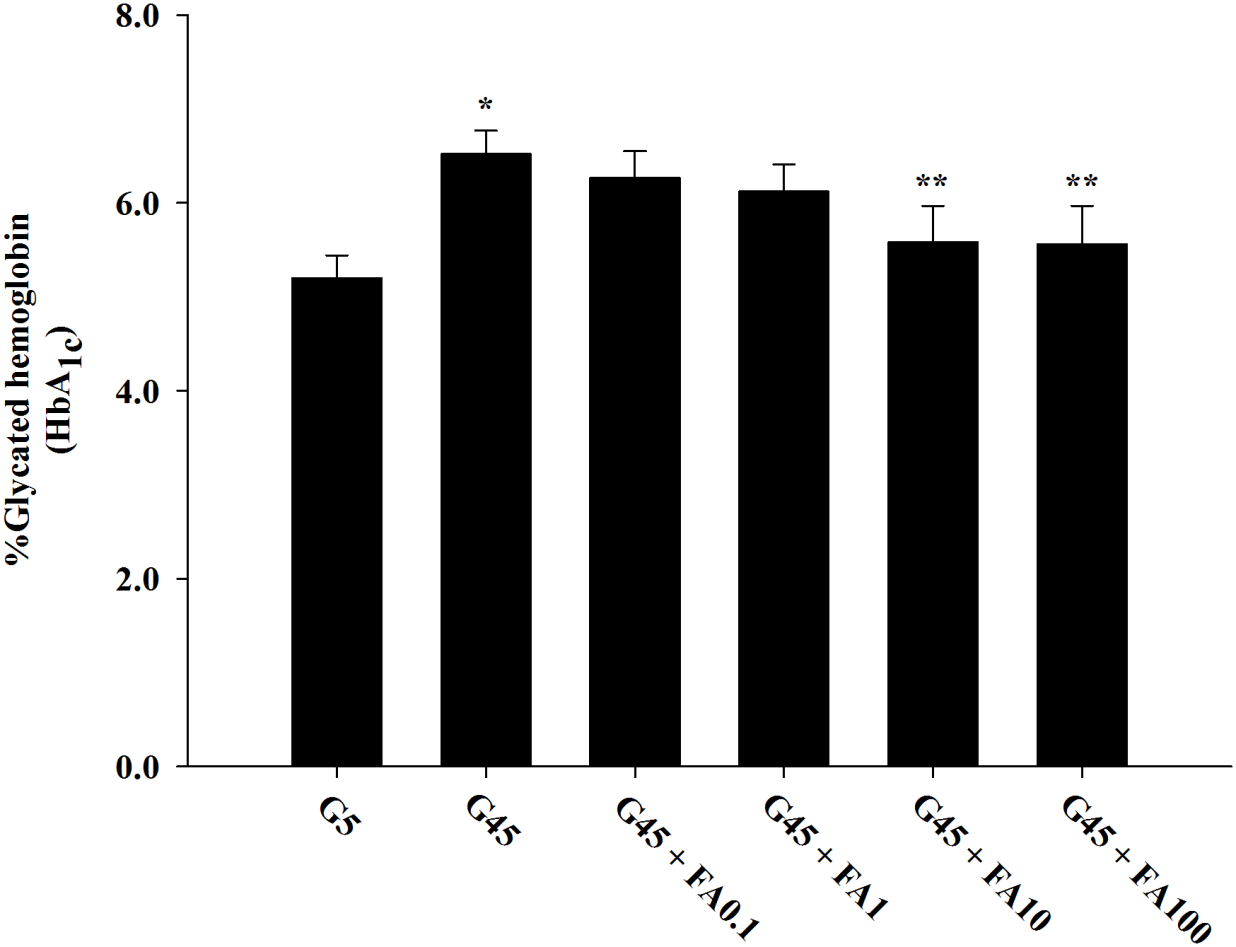
The effects of ferulic acid (0.1–100 μM) on protein glycation (glycated hemoglobin or HbA_1c_) in erythrocytes treated with 45 mM glucose. The results are expressed as mean±SEM (*n* = 6). *p<0.05 compared to 5 mM glucose (G5) and **p<0.05 compared to 45 mM glucose (G45) treatments.

The effects of ferulic acid on Amadori product in erythrocytes treated with high glucose are shown in Fig 2. The results demonstrated that the levels of HbA_1c_ significantly increased about 1.26-fold with 45 mM glucose compared to erythrocytes treated with 5 mM glucose. This increase was significantly inhibited by addition of ferulic acid (10 and 100 μM) about 14.84% and 15.14%, respectively. There were no differences in the levels of HbA_1c_ from erythrocytes treated with 45 mM glucose and ferulic acid at concentrations of 0.1 and 1 μM.

### Effects of ferulic acid on glucose utilization

**Fig 3:**
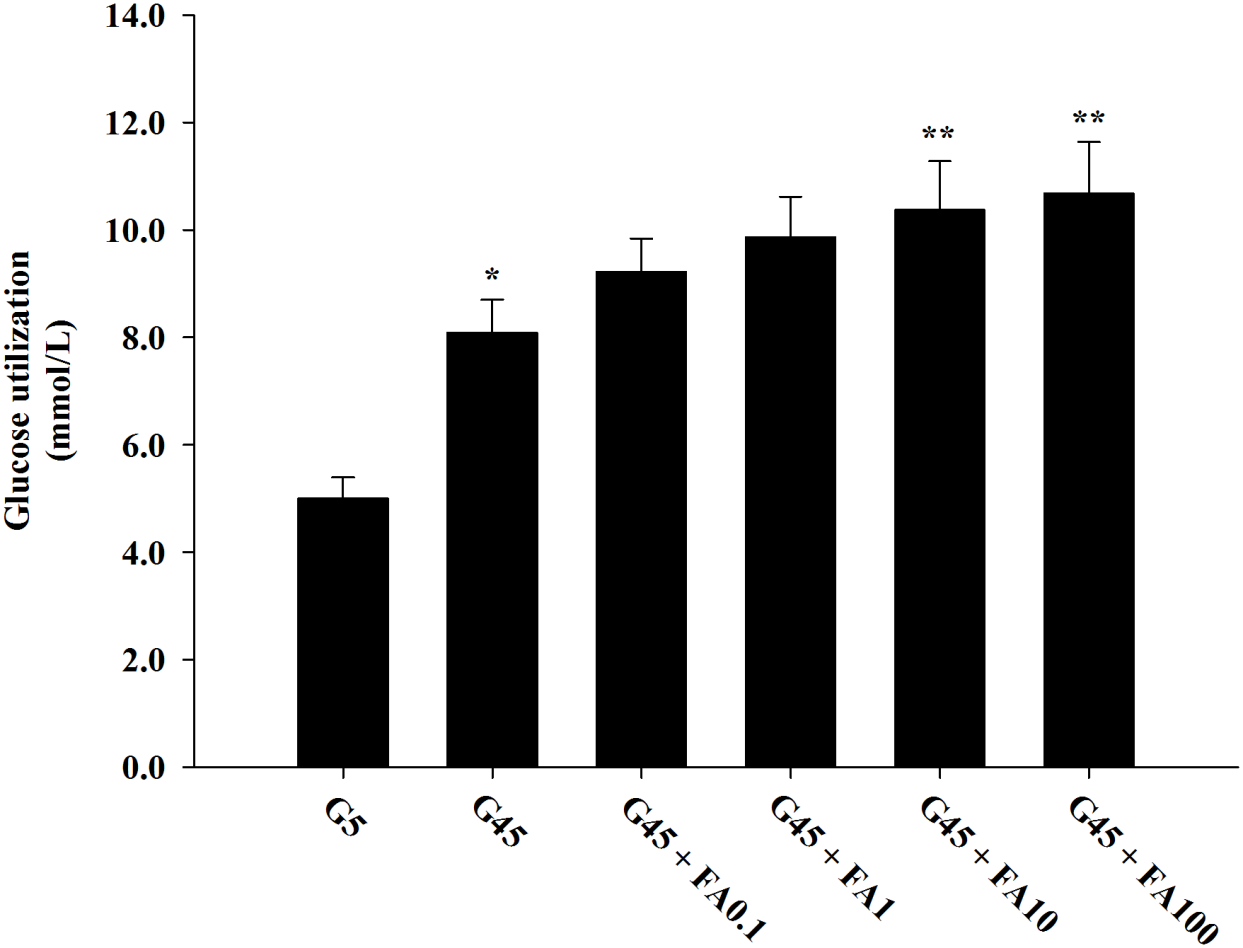
The effects of ferulic acid (0.1–100 μM) on glucose utilization in erythrocytes treated with 45 mM glucose. The results are expressed as mean±SEM (*n* = 6). *p<0.05 compared to 5 mM glucose (G5) and **p<0.05 compared to 45 mM glucose (G45) treatments.

In Fig 3, the effects of ferulic acid on glucose utilization in erythrocytes treated with glucose during 24 h are shown. A significant enhancement in glucose utilization was observed with 45 mM glucose compared to 5 mM glucose. Ferulic acid (0.1–100 μM) with 45 mM glucose caused a gradual increase in glucose utilization by 16.25%, 25.06%, 32.02%, and 36.47%, respectively. It was very interesting that ferulic acid at 10 and 100 μM concentrations significantly increased glucose utilization.

### Effects of ferulic acid on lipid peroxidation

**Fig 4:**
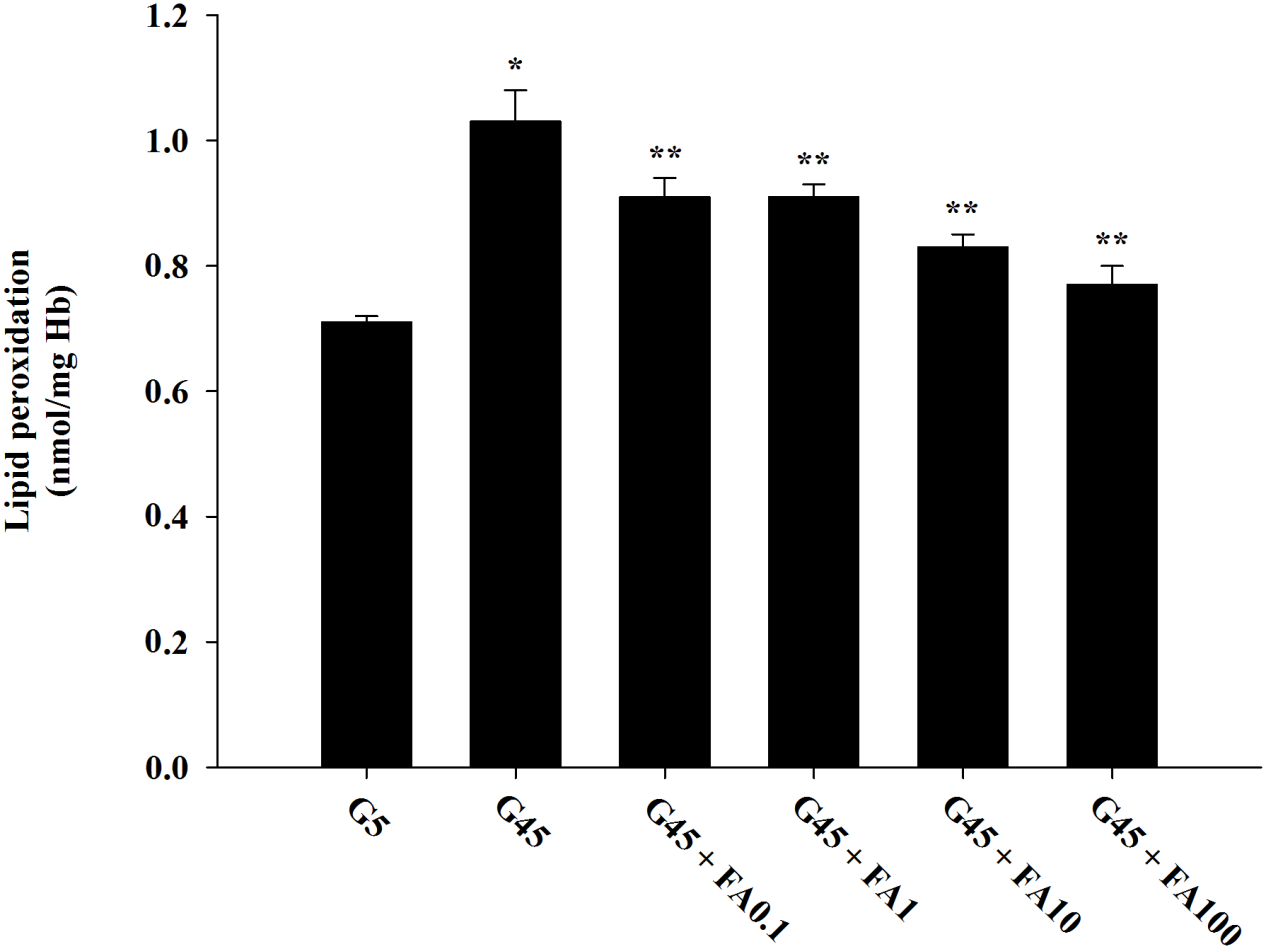
The effects of ferulic acid (0.1–100 μM) on lipid peroxidation in erythrocytes treated with 45 mM glucose. The results are expressed as mean±SEM (*n* = 6). *p<0.05 compared to 5 mM glucose (G5) and **p<0.05 compared to 45 mM glucose (G45) treatments.).

The effects of ferulic acid on lipid peroxidation in erythrocytes treated with glucose are shown in Fig 4. The levels of lipid peroxidation with 45 mM glucose were 1.45-fold higher than with 5 mM glucose. The addition of ferulic acid (0.1–100 μM) with 45 mM glucose significantly decreased lipid peroxidation (11.51%-23.50%) compared to erythrocytes treated with 45 mM glucose alone.

### Effects of ferulic acid on Na^+^/K^+^-ATPase activity

The effects of ferulic acid on Na^+^/K^+^-ATPase activity in erythrocyte treated with glucose are shown in Table 1. A significant reduction in Na^+^/K^+^-ATPase activity was observed with 45 mM glucose (28.33%) compared to 5 mM glucose. The addition of 10 and 100 μM ferulic acid reversed some of the inhibitory effect of high glucose on Na^+^/K^+^-ATPase activity (14.13% and 22.81%, respectively).

**Table 1:** The effects of ferulic acid (0.1–100 μM) on Na^+^/K^+^-ATPase activity in erythrocytes treated with 45 mM glucose.

Treatment	Na^+^/K^+^-ATPase activity (nmol Pi/mg protein/h)
G5	`305.27±5.65
G45	218.48±5.77*
G45 + FA0.1	227.40±6.37
G45 + FA1	235.60±18.20
G45 + FA10	247.89±4.69**
G45 + FA100	267.55±4.37**

The results are expressed as mean±SEM (*n* = 6). *p<0.05 compared to 5 mM glucose (G5) and **p<0.05 compared to 45 mM glucose (G45) treatments.

### Effects of ferulic acid on phosphatidylserine exposure

**Fig 5:**
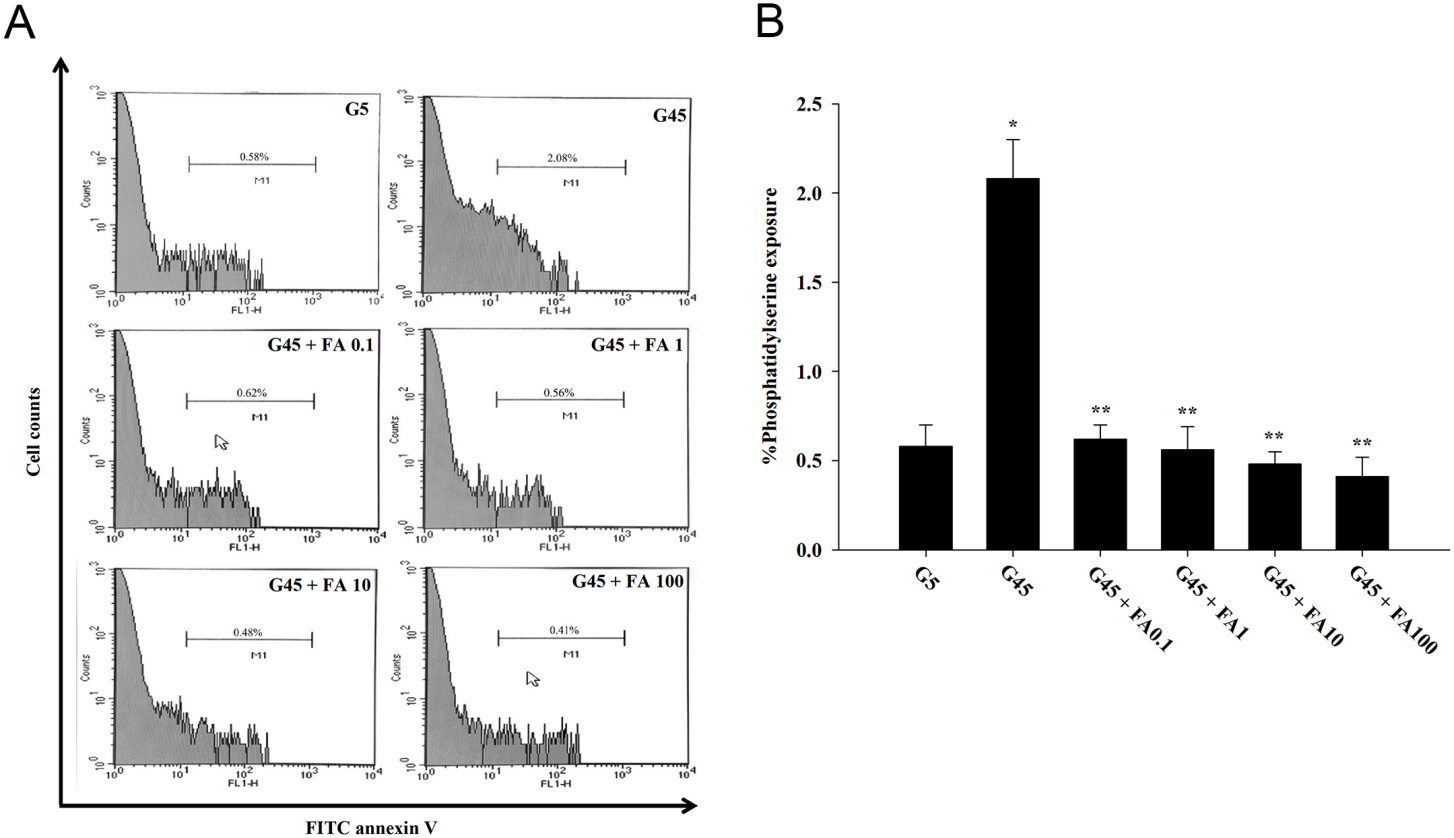
The effects of ferulic acid (0.1–100 μM) on phosphatidylserine exposure (A) and their percentages (B) in erythrocytes treated with 45 mM glucose. The results are expressed as mean±SEM (*n* = 6). *p<0.05 compared to 5 mM glucose (G5) and **p<0.05 compared to 45 mM glucose (G45) treatments.

The effects of ferulic acid on phosphatidylserine exposure in erythrocytes treated with glucose are shown in Fig 5A and 5B. A significant increase in the percentages of phosphatidylserine exposure was observed with 45 mM glucose (3.87-fold) compared to 5 mM glucose. Ferulic acid (0.1, 1, 10, and 100 μM) in erythrocytes treated with 45 mM glucose decreased phosphatidylserine exposure by 68.62%, 70.77%, 74.92%, and 80.91%, respectively, compared to 45 mM glucose alone.

## Discussion

The long-term effect of chronic hyperglycemia contributes to the development of pathogenesis associated with diabetes. In erythrocytes, exposure to high glucose results in increased ROS from auto-oxidation leading to protein glycation [18]. Normally, erythrocytes uptake glucose from the extracellular fluid through glucose transporter-1 (GLUT-1), which undergoes glycolysis to produce ATP and pyruvate [25]. In the absence of mitochondria and oxidative metabolism, pyruvate is reduced to lactic acid *via* anaerobic glycolysis [26]. It is known that glucose utilization by erythrocytes can be increased during hyperglycemia [18]. Elevated extracellular glucose induces glucose toxicity and oxidative stress through auto-oxidation and formation of protein glycation [5,18]. It also increases glucose utilization and HbA_1C_ levels [18]. In the present study, high glucose concentration was used to demonstrate its ability to induce protein glycation in a short time period, similar to other *in vitro* studies [18,21]. Interestingly, ferulic acid increased glucose utilization, thereby decreasing intracellular glucose and inhibiting HbA_1c_ formation under high glucose condition. The precise mechanism by which ferulic acid increases glucose utilization remains unknown. Chang *et al*. reported that cinnamic acid derivatives might play an important role in the stimulation of glucose uptake to improve its utilization in C2C12 cells [27]. The current findings suggest that the increased glucose utilization by ferulic acid might be involved in the activation of glucose uptake in human erythrocytes. In the same cell type, reactive oxygen species (ROS) are produced during high glucose exposure through auto-oxidation and protein glycation [28,29]. ROS causes oxidative degradation of biological molecules especially lipid membrane resulting in cell damage [21]. Lipid peroxidation is widely used as a marker for membrane oxidative damage in other cells [5,18,28,29]. Similar findings are observed in erythrocytes treated with high glucose [5,18,28,29]. An increase in erythrocyte membrane lipid peroxidation is observed in diabetic patients [30]. Considerable interest has been given to antioxidants due to their ability to prevent protein glycation and membrane lipid peroxidation and based on our findings, ferulic acid has similar properties.

Na^+^/K^+^-ATPase pump is an integral membrane protein that plays a major role in the regulation of Na^+^ and K^+^ gradients between extracellular and intracellular space by promoting Na^+^ efflux and K^+^ influx [31]. Therefore, inhibition of Na^+^/K^+^-ATPase pump can affect a number of cellular processes and function [32]. There is evidence indicating that fructose- and methylglyoxal-induced glycation causes the impairment of Na^+^/K^+^-ATPase activity [33]. Down-regulation of Na^+^/K^+^-ATPase pump is observed in streptozotocin-induced diabetic rats [34–37]. Type 1 and 2 diabetic patients often have reduced Na^+^/K^+^-ATPase activity [38,39]. This condition is also seen in diabetic neuropathy [40]. Our findings that erythrocytes treated with high glucose have reduced Na^+^/K^+^-ATPase activity is consistent with previous studies [5,18,28]. Phosphatidylserine is one of four major phospholipids located in the plasma membranes of mammalian cells. It comprises 8–15% of the total phospholipid content [41]. Normally, phosphatidylserine is present in the inner layer of the plasma membrane and facilitates protein binding at the endofacial surface [40]. In addition, it forms an important cofactor for Na^+^/K^+^-ATPase pump [40]. Hyperglycemia-induced oxidative stress, cell damage, and apoptosis cause the exposure of phosphatidylserine into the outer layer of the erythrocyte membrane [41]. Furthermore, erythrocytes from diabetic patients have membrane phospholipid asymmetry with increased surface exposure of phosphatidylserine [42]. This is suggested to facilitate erythrocyte adhesion to the vascular wall [43] and further contributing to thrombosis and microcirculation impairment [44–46]. In our study, erythrocytes treated with high glucose exhibited phosphatidylserine exposure that was consistent with previous findings [47,48]. These results indicate that ferulic acid improves hyperglycemia-induced impairment of Na^+^/K^+^-ATPase activity and decreases the levels of phosphatidylserine exposure in erythrocytes.

In conclusion, we demonstrated that ferulic acid is capable of improving the effects of hyperglycemia on protein glycation and lipid oxidation in erythrocytes. Ferulic acid also increased glucose consumption and Na^+^/K^+^-ATPase activity while reducing phosphatidylserine exposure. These results provide a better understanding of the mechanism by which ferulic acid may help prevent cellular dysfunction and vascular complications associated to diabetes.
